# Transitions between contraceptive states among women aged 15–49 Years in Guinea

**DOI:** 10.1016/j.puhip.2026.100838

**Published:** 2026-07-28

**Authors:** Sidiki Kaba, Alexandre Avdeev

**Affiliations:** Université Paris 1 Panthéon Sorbonne, CRIDUP, Paris, France

**Keywords:** Contraceptive trajectories, State transitions, Contraceptive calendar, Recurrent events, Demographic and health survey, Guinea

## Abstract

**Objectives:**

This study examined transitions between contraceptive states within women's contraceptive trajectories and identified the factors associated with contraceptive continuation, method switching, and discontinuation.

**Study design:**

The study used contraceptive calendar data from the 2018 DHS Guinea. A woman-month event dataset was constructed to reconstruct contraceptive and reproductive histories over the five years preceding the survey.

**Methods:**

Transitions between contraceptive states – non-use of contraception, use of traditional methods, use of modern methods, pregnancy, and pregnancy outcomes – were modelled as recurrent events. To account for multiple transitions experienced by the same woman over time, the Prentice–Williams–Peterson gap-time survival model was applied.

**Results:**

The probability of transitioning to another contraceptive state was estimated at 1.0% among women not using contraception, 1.6% among users of traditional methods, and 2.7% among users of modern methods. The probability increased to 7.5% during pregnancy and reached 61.1% following pregnancy outcomes. Transition probabilities varied only marginally by age, ranging from 23.6% among women aged 15–24 years to 23.0% among those aged 25–34 years and 22.3% among women aged 35–49 years. Similar patterns were observed across educational attainment and household wealth categories. By contrast, exposure to family planning messages and place of residence were not significantly associated with contraceptive transitions.

**Conclusion:**

Contraceptive transitions are primarily driven by reproductive events rather than by socioeconomic characteristics. These findings emphasise the importance of strengthening family planning interventions at key stages of the reproductive life course. However, the findings should be interpreted with caution, as the analysis did not account for sociocultural norms, the availability of family planning services, and the characteristics of local health systems. In addition, the retrospective contraceptive calendar data are subject to recall bias.

## 1 Introduction

According to estimates from the United Nations Population Division, the total fertility rate (TFR) in Guinea stood at 4.22 children per woman in 2023, compared with 2.25 worldwide. At this level of fertility, the country records an average of 1336 births per day, equivalent to approximately 56 births per hour. [1]

Compared with fertility trends observed elsewhere, Guinea's current fertility level is similar to that experienced by France nearly a century ago. It is also comparable to the levels recorded in Seychelles around 50 years ago and in Tunisia, Algeria, Morocco, Botswana, Namibia and Cabo Verde approximately 30 years ago.

Despite the family planning programmes implemented over recent decades, the average number of children a woman is expected to have over her reproductive lifetime remains high in Guinea. This situation is of particular concern because the existing literature consistently identifies family planning as one of the most effective interventions for reducing fertility. [[2], [3], [4], [5], [6]] By enabling women to space and limit births according to their reproductive intentions, family planning is expected to contribute substantially to fertility decline and is widely recognised as a cornerstone of reproductive health [5,6]. However, both the demand for contraception and the level of unmet need remain high in Guinea, suggesting that the anticipated benefits of family planning programmes have not yet been fully realised and highlighting the need to strengthen and expand these interventions. [7,8]

Several barriers limit contraceptive use. These include religious beliefs, cultural norms, myths, misinformation, fear of side effects, and negative attitudes toward contraception. [[9], [10], [11], [12], [13], [14]] Some women perceive certain contraceptives as causing infertility [15,16] or reducing sexual pleasure due to vaginal dryness. [17] In addition, experiences with side effects and rumours may discourage both continued contraceptive use and the adoption of alternative methods. [5,10,11,[18], [19], [20], [21], [22], [23], [24]]

Contraceptive use is also shaped by gender norms and relationship dynamics. [25] Spousal disapproval and fear of marital conflict or dissolution may influence women's contraceptive decisions. [2,21,23,26] Some men believe that women who use contraception may become more attractive to other men and engage in infidelity, [17,19] although other studies report no such association. [27] In some cases, increased marital stability may lead couples to discontinue certain modern methods. [[28], [29], [30]]

Women may also discontinue contraceptive use when they wish to conceive. [9,24,27,[31], [32], [33]] After achieving their reproductive goals, some switch from modern to traditional methods to avoid perceived side effects associated with hormonal or chemical contraceptives. [18,28,32,34]

In addition, women's contraceptive trajectories are influenced by the availability of contraceptive methods, the organization of services, the role of healthcare workers, and the quality of health infrastructure. [7,26,[35], [36], [37], [38]] Staff shortages and long waiting times in health facilities may also limit access to family planning services. [7,36]

Despite widespread knowledge and availability of modern contraceptive methods, the prevalence of unintended pregnancies remains high, reflecting the complexity of contraceptive practices. [3] Studying the dynamics of women's contraceptive trajectories therefore provides valuable insights into how effectively contraceptive services respond to the family planning needs of women and couples. [39]

This study uses the contraceptive calendar from the 2018 Demographic and Health Survey to construct a woman–month event dataset for women of reproductive age. The calendar provides detailed information on the start and end dates of contraceptive use, reasons for discontinuation, and reproductive events such as pregnancy, termination of pregnancy, childbirth, and use of modern, traditional, or no contraception. As these events are multiple and recurrent, several statistical approaches have been proposed in the literature to analyse them, including the Andersen–Gill (AG), Prentice–Williams–Peterson (PWP), Wei–Lin–Weissfeld (WLW), and frailty models.

## 2 Methods

### 2.1 Data

The data used in this study are drawn from the contraceptive calendar of the 2018 Demographic and Health Survey (DHS) conducted in Guinea. The DHS program, primarily funded by the United States Agency for International Development (USAID), has been implemented since 1984 by ICF in collaboration with several partners, including PATH, Avenir Health, Johns Hopkins Center for Communication Programs, Vysnova, Blue Raster, and EnCompass LLC. [40]

The DHS program collects nationally representative data using standardised questionnaires, ensuring comparability across countries and survey rounds. These questionnaires are periodically reviewed and updated to improve data quality. The survey cover a wide range of topics, including marriage, fertility, mortality, family planning, reproductive health, child health, nutrition, and HIV/AIDS. The target population is women of reproductive age (15–49 years). The survey employs four main questionnaires: household, biomarker, woman, and man questionnaires. [40]

### 2.2 Variables

Several sociodemographic variables were included in the analysis. Women's age was computed by subtracting the Century Month Code (CMC) of birth from the CMC of the survey date and was categorised into three groups: 15–24 years, 25–34 years, and 35–49 years. The place of residence was retained as recorded in the survey (urban or rural).

Educational attainment was recoded into three categories: no education, primary education, and secondary or higher education. Household wealth was derived from the DHS wealth index. The first two quintiles were combined into a “poor” category, the middle quintile was retained as “middle,” and the upper two quintiles were grouped into a “rich” category.

Exposure to family planning information was measured using four sources: newspapers or magazines, radio, television, and mobile phones. These variables were retained in their original binary form, indicating whether women had been exposed to family planning messages through each medium.

### 2.3 Event history analysis

The contraceptive calendar provides retrospective monthly information on women's reproductive and contraceptive histories over the five years preceding the survey. It consists of three main columns: the first (vcal_1) records contraceptive use and pregnancies; the second (vcal_2) indicates reasons for contraceptive discontinuation; and the third (vcal_3), where available, provides information on marital status.

The calendar data were first reorganised into monthly variables. Episodes were then identified by detecting changes in the contraceptive status recorded in the calendar. For each month, variables were created to capture contraceptive method use, non-use of contraception, pregnancy, pregnancy termination, or childbirth, as well as the reason for discontinuation.

The dataset was subsequently reshaped into a woman–month structure in long format. Months occurring after the interview date were excluded, and a Century Month Code (CMC) was generated for each observation. Each monthly record was then transformed into an event-based observation, including the start date (ev900), end date (ev901), and event duration (ev901a).

The event codes describing contraceptive use and reproductive outcomes were converted from string to numeric format. Contraceptive methods were coded according to their position in the DHS coding scheme, while pregnancy, pregnancy termination, and childbirth were coded as 83, 82, and 81 respectively. Missing or unknown values were appropriately coded.

Previous, current, and subsequent states were then identified and recoded into six categories: non-use, traditional method use, modern method use, pregnancy, pregnancy termination, and childbirth. The duration of each episode was calculated to construct sequences of contraceptive transitions.

Given the recurrent nature of these events, several survival models for repeated events were considered, including the Andersen–Gill (AG), the Prentice–Williams–Peterson models with gap time (PWP-GT) and total time (PWP-TT), and the Wei–Lin–Weissfeld (WLW) model. Previous studies suggest that the Prentice–Williams–Peterson gap-time model performs robustly across different sample sizes, distributional assumptions, and levels of censoring. [41] For this reason, the Prentice–Williams–Peterson time-interval (PWP-GT) model was selected for the analysis.

Finally, the analyses accounted for sampling weights and the complex survey design of the DHS. This approach ensured the representativeness of the results and the reliability of the estimates, given that DHS surveys are based on a stratified two-stage cluster sampling design.

## 3 Results

### 3.1 Contraceptive method

Women not using any contraceptive method served as the reference group. Women using traditional methods did not differ significantly from non-users, with transition probabilities of 1.0% and 1.6%, respectively. By contrast, the use of modern contraceptives significantly increased the instantaneous risk of transition (HR = 2.684; 95% CI: 2.030–3.549), corresponding to a probability of 2.7% (Table 1).

**Table 1:** Estimation results of the Prentice–Williams–Peterson time interval (PWP-GT) model.

	Haz. ratio	[95% conf. interval]		Marginal effects
		lb	ub	
Contraceptive pathway				
None	Ref.			1.015
Traditional	1.599*	0.992	2.577	1.622
Modern	2.684 ***	2.030	3.549	2.724
Pregnancy	7.361***	5.245	10.329	7.471
Termination of pregnancy	60.177***	34.093	106.220	61.079
Childbirth	60.210***	34.111	106.276	61.111
Woman's age				
15-24	Ref.			23.562
25-34	0.975ns	0.943	1.008	22.971
35-49	0.947***	0.912	0.982	22.302
Education level				
No level	Ref.			22.871
Primary	0.963**	0.930	0.997	22.017
Secondary+	1.058**	1.005	1.114	24.197
Message received by radio				
No	Ref.			22.971
Yes	1.000ns	0.969	1.032	22.970
Message received via television				
No	Ref.			23.011
Yes	0.984ns	0.925	1.048	22.651
Message received by the newspaper				
No	Ref.			22.978
Yes	0.986ns	0.848	1.145	22.651
Message received by phone				
No	Ref.			22.987
Yes	0.955ns	0.792	1.152	21.959
Living environment				
Urban	Ref.			22.804
Rural	1.011ns	0.974	1.051	23.062
Standard of living				
Poor	Ref.			22.471
Average	1.012ns	0.975	1.050	22.730
Rich	1.050***	1.014	1.088	23.598

ns p < 1, *p < 0.10, **p < 0.05, ***p < 0.01.

### 3.2 Family planning messages

Exposure to family planning messages showed no significant effect on contraceptive transitions. These messages are disseminated through newspapers, radio, television, and mobile phones (Table 1).

### 3.3 Reproductive events

Pregnancy was associated with a sevenfold higher instantaneous risk of transition (HR = 7.361; 95% CI: 5.245–10.329), with a transition probability of 7.5%. Following pregnancy outcomes such as termination (HR = 60.177; 95% CI: 34.093–106.220) or childbirth (HR = 60.210; 95% CI: 34.111–106.276), women experienced a marked acceleration in contraceptive transition, with probabilities of approximately 61% (Table 1).

### 3.4 Sociodemographic factors

Age differences were modest. Women aged 25–34 years had a slightly lower risk of transition compared with those aged 15–24 years, although this difference was not statistically significant. However, women aged 35–49 years showed a significantly lower instantaneous risk of transition (HR = 0.947; 95% CI: 0.912–0.982). The corresponding transition probabilities were 23.6%, 23.0%, and 22.3% for women aged 15–24, 25–34, and 35–49 years, respectively (Table 1).

Educational attainment also influenced transitions. Women with primary education had a slightly lower risk of moving to the next state (HR = 0.963; 95% CI: 0.930–0.997), whereas those with secondary or higher education had a higher risk (HR = 1.058; 95% CI: 1.005–1.114), with transition probabilities of 22.9%, 22.0%, and 24.2%, respectively (Table 1).

Women living in wealthier households had a slightly higher risk of transition compared with those in poorer households (HR = 1.050; 95% CI: 1.014–1.088). No significant difference was observed for women in middle-income households (Table 1).

Finally, place of residence was not significantly associated with contraceptive transitions. Thus, contraceptive transitions remain relatively similar among women living in rural and urban areas (Table 1).

## 4 Discussion

The findings indicate that contraceptive trajectories differ according to the type of contraceptive method used. Women relying on traditional methods generally exhibit more stable contraceptive trajectories, whereas users of modern methods experience more dynamic patterns characterised by more frequent transitions. This finding is consistent with previous studies reporting higher rates of contraceptive discontinuation and method switching among users of modern methods, particularly injectable contraceptives and intrauterine devices. [4,42] Such patterns may reflect the greater flexibility of modern methods, changes in reproductive intentions, or dissatisfaction associated with side effects.

Reproductive events emerged as the strongest determinants of contraceptive trajectories. Pregnant women were significantly more likely to experience transitions in their contraceptive histories, supporting previous evidence that pregnancy represents a critical period during which women reconsider their future contraceptive intentions. Many women plan to initiate contraception after childbirth, opting for methods such as implants, intrauterine devices, oral contraceptives or sterilisation. [37]

Similarly, childbirth and pregnancy termination constituted major turning points in women's contraceptive trajectories. These reproductive events were frequently followed either by another pregnancy or by the adoption of a modern or traditional contraceptive method. Previous studies have shown that, after childbirth, some women adopt contraception to space subsequent births, whereas others postpone contraceptive decision-making or select a method based on counselling received from healthcare professionals and their own postpartum experiences. [37,38,43] Together, these findings emphasise that contraceptive behaviour evolves throughout the reproductive life course and is strongly influenced by women's reproductive experiences.

Although age-related differences were relatively modest in the present study, age remains an important determinant of contraceptive behaviour. Women aged 35–49 years were generally less likely to change contraceptive methods than younger women, whereas younger women were more likely to initiate or switch contraceptive methods, particularly short-acting methods such as oral contraceptive pills and condoms. Previous studies have likewise shown that contraceptive use tends to decline with increasing age. [21,25,29,42,44,45]

Educational attainment was also associated with contraceptive trajectories. Women with secondary or higher education experienced more rapid transitions than women with no formal education. This finding is consistent with previous research indicating that education improves knowledge of contraceptive methods and increases the likelihood of adopting or switching contraception. [26,29,34,44,[46], [47], [48]] Better-educated women are also more likely to adopt health-promoting behaviours and to use long-acting or permanent contraceptive methods. [19,24,32,49,50]

Household economic status also influenced contraceptive trajectories. Women from wealthier households were more likely to adopt contraceptive methods and to experience dynamic contraceptive trajectories than those from poorer households. [29,51] Conversely, women from disadvantaged socioeconomic backgrounds were more likely to discontinue contraceptive use because financial constraints limited their access to family planning services. [13,14,18,34,35,47] These findings reinforce the importance of reducing financial barriers to improve equitable access to contraception.

By contrast, neither place of residence nor exposure to family planning messages was significantly associated with transitions in contraceptive trajectories. Nevertheless, previous studies suggest that women living in rural areas often experience higher rates of contraceptive discontinuation and receive less partner support for family planning than women residing in urban areas. [12,33,47,48] Likewise, although no significant association was observed in the present study, access to accurate and high-quality family planning information remains essential for supporting informed contraceptive decision-making. In particular, counselling delivered during antenatal care, childbirth and the postpartum period has been shown to improve contraceptive uptake, continuation and the management of method-related side effects. [14,22,52]

Overall, the findings demonstrate that reproductive events – particularly pregnancy, childbirth and pregnancy termination – are the primary drivers of transitions within women's contraceptive trajectories. These life events represent critical turning points that accelerate movement between contraceptive states, whereas sociodemographic and economic characteristics, together with exposure to family planning messages, exert a comparatively smaller influence. Collectively, these findings suggest that contraceptive decision-making is closely shaped by women's reproductive experiences and evolves throughout the reproductive life course.

From a policy perspective, these findings highlight the importance of strengthening family planning interventions at key stages of the reproductive cycle, particularly during pregnancy, childbirth and the postpartum period. Integrating comprehensive family planning counselling and contraceptive services into antenatal, delivery and postnatal care could facilitate both the uptake and continuation of contraceptive methods that are appropriate to women's reproductive intentions and changing needs. Such an integrated approach would enable health systems to provide more responsive and woman-centred family planning services.

This study makes an important contribution to the literature by examining contraceptive trajectories as dynamic processes rather than isolated contraceptive events. The use of the Prentice–Williams–Peterson recurrent event regression model allowed repeated transitions between contraceptive states to be analysed, providing a more comprehensive understanding of contraceptive behaviour across women's reproductive lives.

Nevertheless, several limitations should be acknowledged. The analysis did not account for contextual factors such as sociocultural norms, partner characteristics, the availability and quality of family planning services, or local health system characteristics, all of which may influence contraceptive trajectories. In addition, the retrospective contraceptive calendar is subject to recall bias and dating errors that may have affected the accuracy of the reconstructed contraceptive histories. Future studies incorporating contextual variables and prospective longitudinal data would provide a more comprehensive understanding of the mechanisms underlying contraceptive trajectories.

Taken together, these findings demonstrate that reproductive events play a central role in shaping women's contraceptive trajectories. By adopting a life-course perspective and modelling repeated contraceptive transitions, this study provides robust evidence to inform reproductive health policies and family planning programmes while contributing to the advancement of research in reproductive demography.

## Ethical statement

Not applicable. The study was based on the secondary analysis of anonymised Demographic and Health Survey (DHS) data.

## Funding

None.

## Declaration of competing interest

No conflict of interest.
